# Development and operationalization of a data framework to assess quality of integrated diabetes care in the fragmented data landscape of Belgium

**DOI:** 10.1186/s12913-022-08625-8

**Published:** 2022-10-18

**Authors:** Buffel Veerle, Danhieux Katrien, Philippe Bos, Remmen Roy, Van Olmen Josefien, Wouters Edwin

**Affiliations:** 1 Department of Sociology, University of Antwerp, Antwerp, Belgium; 2 Department of family medicine and population health, University of Antwerp, Antwerp, Belgium

**Keywords:** Primary care, Routinely Collected Data, Quality of Healthcare, Type 2 Diabetes, Integrated Delivery Systems

## Abstract

**Background:**

To assess the quality of integrated diabetes care, we should be able to follow the patient throughout the care path, monitor his/her care process and link them to his/her health outcomes, while simultaneously link this information to the primary care system and its performance on the structure and organization related quality indicators. However the development process of such a data framework is challenging, even in period of increasing and improving health data storage and management. This study aims to develop an integrated multi-level data framework for quality of diabetes care and to operationalize this framework in the fragmented Belgium health care and data landscape.

**Methods:**

Based on document reviews, iterative working group discussions and expert consultations, theoretical approaches and quality indicators were identified and assessed. After mapping and assessing the validity of existing health information systems and available data sources through expert consultations, the theoretical framework was translated in a data framework with measurable quality indicators. The construction of the data base included sampling procedures, data-collection, and several technical and privacy-related aspects of linking and accessing Belgian datasets.

**Results:**

To address three dimensions of quality of care, we integrated the chronic care model and cascade of care approach, addressing respectively the structure related quality indicators and the process and outcome related indicators. The corresponding data framework is based on self-collected data at the primary care practice level (using the Assessment of quality of integrated care tool), and linked health insurance data with lab data at the patient level.

**Conclusion:**

In this study, we have described the transition of a theoretical quality of care framework to a unique multilevel database, which allows assessing the quality of diabetes care, by considering the complete care continuum (process and outcomes) as well as organizational characteristics of primary care practices.

**Supplementary Information:**

The online version contains supplementary material available at 10.1186/s12913-022-08625-8.

## Background

Type 2 Diabetes (T2D) is one of the leading causes of death in the world with 3.7 million deaths/year [1]. In Belgium, 6.1% of the population is diagnosed with diabetes [2]. Effective interventions for prevention and control are available and are relatively straightforward from a technical point of view. There are already several international [3, 4] and national evidence-based guidelines [5–8] regarding the management of diabetes and the development of quality indicators (QI) [9] to monitor and assess the quality of diabetes care is prioritized by the Organization for Economic Cooperation and Development (OECD) [9]. However, T2D care remains socially and organizationally complex, and successful implementation and follow-up of the guidelines is still not self-evident [10]. It requires lifelong follow-up and self-management along a continuum of care: patients need to be tested, diagnosed, linked to care, treated, followed up, and supported to achieve glycemic control. Unfortunately, a substantial amount of people are lost at each of these steps, leading to sub-optimal uptake of high-quality care [11].

These gaps in the continuum of care are related to both demand (patient) and supply (healthcare organization) side characteristics. At the demand side, research has indicated that people living in socio-economically vulnerable conditions are significantly more likely to be lost alongside the continuum of care [11]. This contributes to the growth of health inequalities that health systems were designed to address but failed to do [12]. At the supply side, differences exist between providers in how they treat their patients and how they organize their practices, especially in primary care, where the biggest volume of T2D patients is treated [13, 14]. Therefore, assessing the quality of T2D care, investigating differences in this quality between patient groups and different types of primary care practices and explaining these differences are highly relevant.

Following Donabedian’s landmark model [15–18], quality of care has three dimensions: 1) structure; 2) process; and 3) outcomes. The first dimension [15] is about how care is organized. It refers to the elements that form the basis of the healthcare system, including the accessibility of care facilities [19, 20], an adequate mix of human resources [21], up-to-date equipment [22], a well-working health information system (HIS), and integrated policies. The second dimension addresses the questions on the medical interaction (at a technical and interpersonal level). It refers to the completeness, continuity, and functional quality of activities for diagnosis and treatment. The third dimension covers the intermediate health outcomes and/or the end results of the healthcare or intervention. These are rather indirectly related to the care provider’s actions and the organization of care, and are much more influenced by other factors such as patient characteristics and the environmental context [23]. These three dimensions are especially relevant for the quality of chronic care, such as diabetes care [13, 24, 25].

In order to get a comprehensive overview of the multidimensional quality of care for a chronic disease such as T2D, one needs complex data –i.e. data on structure, process and outcome of a continuum of care. Ideally one should follow the patient (demand side) along this care continuum to assess the process and outcomes of primary healthcare. In addition, information on the organization of healthcare (supply side) is required to assess the performance of structure related QIs. These data at patient and primary care practice level should also be linkable to further investigate the impact of structure related factors on the performance of the care process and outcomes.

In the last decades, and especially since the St Vincent Declaration in 1989 [26] –a joint effort to reduce the burden of diabetes by listing measures to be taken by nations and organizations– a broad consensus on the importance of reliable health information has grown, together with a proliferation of individual health information registration and storage. This gave rise to a series of initiatives aimed to establish and improve monitoring and control systems for quality assurance of diabetes health care provision (e.g. DiabCare [27], EUDIP [28], EUCID [29], EUBIROD [30], PaRIS [31]). One of its merits is the development of a set of basic indicators for the quality of diabetes care, which are feasible to collect on a national scale and particularly suitable for monitoring and assessing quality of care over time and across health systems. These indicators are, however, mostly unlinked, based on aggregated data, stratifiable to only a few basic patient characteristics (such as, for instance, gender and age) and cover only process and outcome related measures, making them less adequate for investigating differences in quality of care between patient groups and types of primary care practices and to explain these differences. This is important since health systems increasingly are evaluated for their performance to reduce inequity between groups.

The linkage of the three QIs remains thus challenging, also within the recent innovations in HIS. Until now, it has only been done at a small scale (in a few health centers, hospitals or intentionally collected for a study project [21, 32]) and at aggregated levels making it impossible to study quality differences between patient groups and primary care organizations, or in countries with a very well-structured HIS, for example where a comprehensive diabetes register is available [33] (such as in Sweden [34, 35], the UK [36], Denmark [37], and Toronto [38]).

Especially in fragmented healthcare systems with a similarly complex health data landscape, the development process of such a data framework is constrained by conceptual, organizational, technical, and legal barriers. Conceptually, a standardized language of patient health information registration (electronic health records (EHRs)) is still lacking between different registration systems and health services. At the organizational level, these EHRs are often not stored or made accessible at a central database or platform and need to be manually extracted and collected. Or they are only provided at the aggregated level (e.g. primary care practice level, regional level), which may lead to wrong interpretations of observed quality differences, hiding differences in patients’ needs. As alternative to EHRs, several studies rely on health insurance data. In these data, however, outcome related quality information is mostly lacking, as the majority of clinical outcomes are rarely registered for administrative purposes. Many information is thus stored but in a fragmented way without an overall vision, such that discussions on monitoring datasets are preceded by discussions about the relevance, priority, feasibility and validity of the data included in such datasets [39]. This then results in technical, legal and ethical challenges when researchers attempt to get access to the data and to link the datasets (e.g. data matching procedures, data protection issues, etc.) [40].

In this study, we aim to address these challenges by developing and operationalizing a multilevel data framework for the quality of T2D care, in the context of a highly fragmented health system without a national diabetes register [33], as is the case in Belgium. The study addresses the following research questions: 1) How do we measure and link the structure, process and outcomes of integrated diabetes care?; 2) Which data sources are available and what are their strengths and limitations?; and 3) How do we design and operationalize an integrated diabetes care database, allowing quality assessment and the analysis of differences in quality between patients groups and primary care practices.

## Methods

### Study setting

This study is part of a larger research project SCUBY ‘SCale-UP Diabetes and hYpertension care in Belgium, Cambodia and Slovenia’ [41]. The current study is approved by the Ethical Committee of the University Hospital Antwerp (ref. 20/06/069) and all methods were performed in accordance with the relevant guidelines and regulations (Declarations of Helsinki).

In Belgium, healthcare providers and patients enjoy a high degree of autonomy in their choice of service utilization and care provider, which has led to a fragmented system of individualized care [19, 20]. Patients are not obliged to register at one General Practitioner (GP) and GPs can choose their financing system (fee-for-service (FFS) or capitation) and how they organize their practice (solo versus group, multi- versus monodisciplinary). This complexity and high degree of autonomy is also reflected in the Belgian HIS and data landscape. At the primary care level, various EHR systems co-exist and the major aim of these systems is to facilitate medical record-keeping for the GP. Health care professionals only share limited information with each other and the exportation of EHR data to administration or quality control institutions is no routine. A recent development is the incentivization for adequate registration [40] of a limited number of indicators, such as the percentage of people in a diabetes care trajectory or the number of COVID diagnoses [42].

Over the last decade, several efforts to increase harmonization and interoperability in the Belgian health information landscape have been initiated, such as the national electronic health (eHealth) action plan in 2008 and healthdata.be in 2015. eHealth is a federal government agency whose mission is to support a well-organized, mutual electronic service, data transfers and information exchange between all actors in the healthcare sector, with a focus on data security and privacy protection of the patient and the healthcare provider [43]. Since 2018, this online data portal also has a personal health viewer (called ‘mijngezondheid.be’), where patients can consult their health information after registration [44]. Healthdata.be as part of eHealth aims to centralize and improve clinical registries in a new data platform. This platform currently collects data for more than 150 clinical registries, from multiple sources such as primary care facilities, laboratories, and hospitals, and each with multiple information systems or EHR [40]. However, there are still ongoing projects, where the centralization and integration of data at patient and health service level covering different dimensions of health, health care and costs and in a GDPR-proof way, remain challenging.

In contrast to several other European countries, Belgium has no comprehensive diabetes registry [45]. In 2001 the ‘Initiative for Quality improvement and Epidemiology in Diabetes’ (IQED) was launched [46, 47]. This project is based on the principles of Diabcare (a WHO dataset on diabetes) [27] and assesses and monitors the quality of diabetes care through regular data collection among individuals with diabetes who require specialized treatment and are followed-up in hospital-based centers [46, 47]. This quality assurance project also provides individualized feedback, helping the centers to improve their quality of care [. Although this dataset is very useful for a subgroup of insulin-dependent patients, the current study is focusing on integrated care mainly delivered at the primary care level to all (pre) diabetes type 2 patients, including patients with mild symptoms, as especially among this group of patients, early detection and appropriate follow-up is necessary to overcome exaggeration of their symptoms and complaints.

### Study design

**Fig. 1:**
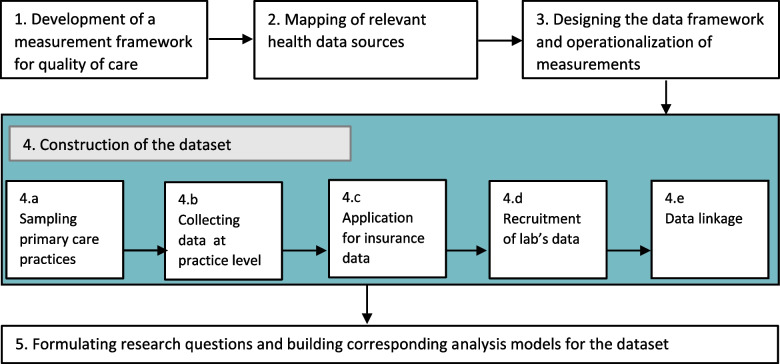
Flowchart of the phased approach of the development of the data framework and quality assessment tool

We used a case study approach by focusing on chronic care for T2D patients, but the proposed methodology and the development of the measurement and multilevel data framework can also be relevant for the assessment of the quality of chronic care in general or other specific types of chronic care. We relied on a phased approach (presented in Fig. 1): phase 1) development of a measurement framework for quality of care; 2) mapping of relevant health data sources; 3) designing the data framework and operationalizing the indicators, 4) construction of the dataset and 5) formulating research questions and building the corresponding analysis models for the dataset.

### Research methods

Phase 1 – We started with a document review on models for integrated chronic care and systems of quality assessment covering and linking the three quality dimension. An explorative review on documents and guidelines for integrated chronic care models and quality assessment instruments was done, using Medline and PubMed databases. The search combined various terms for quality of chronic care and integrated care, dimensions of quality of care, care assessment and quality indicators. In addition, we searched on the websites of governmental entities (e.g. Scientific institute for public health [Sciensano], Belgian Health Care Knowledge Center [KCE], etc.) in Belgium and other international relevant organizations such as the World Health Organization (WHO), the OECD and International diabetes federation (IDF). During a working group discussion with the international SCUBY research team of experts the theoretical frameworks were assessed on their usefulness for our research objectives. The end result of this phase is a measurement framework for quality of care at a theoretical level (Table 1).

**Table 1:** Dimensions of quality of care addressed by the integration of the chronic care model and cascade of care

Dimension of quality of care *(cfr. Donedian’s model)*	Theoretical approach	Measuring tool	Data level
Structure and organization	Chronic care model	ACIC-Sub scores: -Organization -Community linkages -Self-management support -Decision support -Delivery system design -Information systems	-Health system -Primary care practice
Process	Cascade of care approach	CoC bars: -tested -diagnosed -linked to care -taking treatment -followed up,	-Patient (individual level)
Outcomes		-under control	

Phase 2 – Before moving on to the practical implementation of this framework, an inventory was made of all health information systems in Belgium related to the delivery of the integrated care package. This was achieved through qualitative research: we started with an explorative document review to get a first idea of all relevant data bases available in Belgium and thereafter we had seven key informant interviews with responsible people of the largest data providers and with people who develop and manage health information systems: the Flemish general practice-based morbidity registration network (Intego), Sciensano, the largest Belgian health insurance (CM), the Intermutualistic Agency (IMA), a clinical research database in out-of-hours care (iCAREdata) and medical labs. During the interviews, we discussed the characteristics of the datasets (type of data, period, period, target population and representativeness, content, advantages and disadvantages), especially in the light of our research objectives. We were also guided by the key informants to other datasets and projects that are available in Belgium and could be relevant for our research objective. The document review and interviews were used as input for the mapping exercise, which was done to assess the different health information systems – in order to see whether all data required to monitor the integrated care package are available – and the options to connect these systems allowing the linkage of the three dimensions of quality of care (Results presented in Table 2).

**Table 2:** Results of the assessment of the existing health data sources for T2D care

Type	Collected/owned by (part of project)	Period	Population (representativeness)	Content	Strengths	Limitations
**PATIENT SURVEY DATA**						
Health Interview survey (HIS)^a^	Sciensano (Belgian Institute for Health)	Repeated cross-sectional: every 4 years since 1997	Representative sample of the Belgian population (N = +/−10,000 respondents)	medication use, health care use and costs, health behavior (physical activity, diet, smoking, alcohol), BMI, diagnostic information	-Representative for the population -Extensive lifestyle and sociodemographic info	-Small numbers of T2D patients (6,2%) -Self-reported data, recall bias -Selection and sample bias -The most severe and institutionalized patients are excluded -Time lag: data of 2018 was available in 2021 -Cross-sectional data -No clinical data -No information about type of GP-practice
Belgian Health Examination study (BELHES)^b^		Cross-sectional: 2018	Representative subsample of the HIS (N = +/1200 respondents)	blood and urine test, blood pressure measure, BMI	-Clinical data -Can be linked to HIS data	-Small sample (+ same limitations HIS data)
**HEALTH PROFESSIONAL SURVEY DATA**						
Sentinel Network of General Practitioners (surveillance network)^c^	A network of Registered GPs, coordinated by Sciensano	Longitudinal: since 1979 Periodic modules to monitor one or more specific illness problems	A network of 125 practices (their patient population covers 1–1,5% of the Belgian population)	sociodemographics, treatment and morbidity data	-Representative for Belgian GP workforce -Able to study the evolution and epidemiology of certain diseases	-Quality of data strongly depends on the reporting quality of GPs -The most recent T2D module was in 2010
**(REGISTER) DATA**						
Belgian Diabetes Register ^d^	Diabetes Liga	Longitudinal: since 1997	New patients < 40 years old diagnosed with T1D	sociodemographics, clinical data	-Clinical data -Longitudinal	-Only T1D patients (not the target population of this study)
IQED: Initiative for Quality Improvement & Epidemiology in Diabetes ^e^	Hospital based data requested by Sciensano for audit (Surveillance of the convention for diabetes self-regulation)	Repeated cross-sectional retrospective study design (every 18 months): since 2001	Patients in a diabetes care trajectory: +/− 100 diabetic centres treated +/− 120,000 patients -each time 10% of the population is sampled	clinical hospital data, socio-demographics, type of diabetes and complications, diabetes treatment, health examination data	-Clinical data -Focus on quality indicators -Based on principles of DiabCare	-Only type 1 and type 2 diabetic patients treated with 2 or more insulin injections per day (only a small part of the target population of this study)
**PATIENT RECORD DATA**						
Patients records ^f^	GPs in their practices	Longitudinal: period depends on GP-practice	Patient-population of GP	Depending on GP (diagnostic info, health services, medication, health behavior, severity of diseases, comorbidities, familial anamnesis, RISC score, ….)	-Data can be very comprehensive (BMI, blood pressure, waist circumcise, smoking behavior, etc.) -Diagnostic, health care use, medication prescription and clinical data (GPs have easily access to the lab data of their patients)	-Several software systems: no standardized way of registration -Low reporting quality & large differences between practices -Difficult & time-consuming to extract the data (a lot of efforts for GPs and in particular if there is no administrative staff) -Data is not centralized
Primary care registry based on patient records (Electronic Health Record)	Intego data^g^ Computerized morbidity registration network of participating practices.	Longitudinal: since 1999	Patient population of +/− 50 participating practices	Morbidity in primary care; diagnostic data, sociodemographic data, health care and medication data. The data is aimed to perform audits of the primary care.	-Representative for Flemish population -A lot of diagnostic and clinical longitudinal data -Large number of patients	-Currently Intego is in a transition phase and Medidoc (the software) does not longer exist: as a result no recent data is available -Quality of data depends on coding behaviour of clinicians and there is a lot of variation therein between GPs -Data from specialists as well as events that occur in hospital are not fully captured
**HEALTH CLAIMS DATA (administrative)**						
Databases of the Intermutualistic Agency (IMA)^h^: Population database, Health care data &Pharmanet	gathered from the seven Belgian health insurance funds that manage compulsory health insurance	Longitudinal	Entire insured Population data (> 99% of the population)	sociodemographic data, health care and costs data, medication data (all reimbursed medication and health services), hospital visits (duration), etc.	-inexpensive compared to original data collections -population data - detailed health care data -Continuously collected -Standardized data registration -Linkage based on a unique identifier number is possible -Previously used in research on chronic care	-Not collected and designed for scientific purposes: not structured in readily available variables for analyses, -Lack of clinical and diagnostic information -No information about health behavior, BMI, etc. -Time lag: data is available in February year X of Year X – 2
**MEDICAL LAB DATA**						
Data of the Medical laboratories	Laboratories (on request of GPs and specialists)	Depending on the lab	Each lab covers the patient population of several GPs/specialists/ hospitals	Clinical information (type and result value of test)	-Data extraction and linkage based on a unique identifier number is possible -Longitudinal data -Comprehensive clinical information	-Only clinical information −+/− 70 accredited labs -Several Lab information systems (LIS)

Sources: ^a^https://www.sciensano.be/en/projects/health-interview-survey-2018; ^b^https://www.sciensano.be/en/projects/health-examination-survey; ^c^https://www.sciensano.be/en/network-general-practitioners^d^https://www.diabetes.be/belgisch-diabetes-register; ^e^https://www.sciensano.be/en/projects/initiative-quality-improvement-and-epidemiology-diabetes;^f^https://www.ehealth.fgov.be/ehealthplatform/file/view/AWutmy6TnF_Mkwg-mMBj?filename=GP%20documentation%20-8th%20July%20%202019.pdf;^g^https://intego.be/nl/Welkom; ^h^https://ima-aim.be/-Onze-databanken

Phase 3 *–* After our theoretical considerations on how the structure, process and outcomes of integrated diabetes care should be measured (Phase 1) and getting insight in the state of the art regarding relevant databases available in Belgium (Phase 2), the 3rd entailed the practical elaboration of the theoretical framework. It consists of a design process on how to construct a joint database that would allow an integrated analysis of the different data elements for a study population, and the operationalization of the quality measures. Therefore, a document review of the national (Domus Medica [6]) and international guidelines (IDF [3], ADA [4], NICE [8], SIGN [5]) on T2D management and the available set of indicators (QoC OECD set [9], DiabCare set [27]) was performed (in contrast to the review in phase 1 the focus was on the practical implementation). This comprehensive list of guidelines and indicators was systematically assessed on relevance, feasibility, and validity for monitoring the different dimensions of quality of diabetes care in iterative meetings with a working group (results in Table 3). In total three meetings were done with a multidisciplinary working group (existing of general practitioners, epidemiologists, public health researchers, sociologists, and pharmacists) and one specific working group meeting on statistical preparations.

**Table 3:** The operationalization of the Cascade-of-Care

Stage of CoC		Time (year)	Operationalization	Source	Reference	Remarks
1	Tested	x-3 to x-1 (2015–17)	every 3 year a blood test on glucose/HbA1c	IMA	Domus Medica [6], CDC [7], IDF [3]	Domus medica & IDF: from age 40 ideally combined with Findrisc (FINnisch Diabetes risc score) test (but this is not included in the data) CDC: from age 45 IDF: from age 40–45
2	Diagnosed	x-1 (2017)	meeting the inclusion criteria: T2D medication or pre-diabetes pass in selection year (2017) Exclusion criteria: convention Type 1 diabetes and/or prescription insulin pump (only reimbursed for T1D) (in selection year or previous year)	IMA	[48, 49]	Using validated proxies, as we work with insurance data. T2D medication = Metformin, Sulfonylurea, Insulin Pre-diabetes pass = provides a better framework of care for pre-diabetes patients (including reimbursement of yearly four diabetes education consults provided by a dietician, diabetes educator, nurse, pharmacist, or physiotherapist) To exclude as good as possible type 1 diabetes patients, we also have two exclusion criteria.
3	In care	x-1 (2017)	At least one GP visit (in selection year)	IMA	IDF [3]	As for patients in a capitation system GP-visits are not registered, an alternative measure is used for this group: “at least one medication or lab test prescription of a GP in selection year 2017” (as sensitivity analysis: using this indicator also for non-capitation patients and comparing with the other indicator)
4	In treatment	x (2018)	T2D medication in 2018 or, among patients in pre-diabetes trajectory, at least one T2D education or dietician consult	IMA	Domus Medica [6], IDF [3]	For patients in a prediabetes care trajectory an annual consult with a diabetes educator and dietician is reimbursed.
5	Follow up	x to x + 1 (2018–19)		IMA/Lab-data	IDF [3], Domus Medica [6], QoC OECD [9]	Once ‘AND’ (meeting all criteria) and once indicator specific (i.e. % that meets each criteria separately)
			> = 2 HbA1c measurements (at least one in 6 months)			Process indicator of QoC OECD: Percentage of patients with one or more HbA1c tests annually
			annual lipid profile measurement			to prevent additional cardiovascular disease (estimating cardiovascular risk) Process indicator OECD diabetes QoC: LDL cholesterol test annually
			annual microalbuminuria measure			To control kidney function
			annual creatinine measurement (and eGFR calculated)			To detect additional complications (diabetic nephropathy)
			annual food examination			To detect additional complications (neuropathy & foot complications)
			annual consultation by an ophthalmologist		IDF [3], Domus Medica [6], NICE [8], SIGN [5], ADA [4]	To detect additional complications (retinopathy)
6	Under control	x + 1 (2019)	HbA1c < 53 mmol/mol	Lab-data	IDF [3], Domus Medica [6]	Exploring whether we can stratify by ‘totally not under control’; ‘just not under control’; ‘just under control’; ‘well under control’

Phase 4 – The fourth phase entailed the actual construction of the date framework and dataset: data collection and sampling procedure (results in Supplement 1), stakeholder discussions with the data providers (IMA and laboratories) about which data and how they should be delivered (format, transfer mode, etc., see S2), and the design of the data linkage strategy together with data experts of IMA and eHealth (results in S3). It also included ethical approvals and data applications.

Phase 5 – As last, we discuss the analysis opportunities of the data set in a working group discussion with the Belgian SCUBY research team.

## Results

### Phase 1: development of a measurement framework for quality of care

As Donabedian prescribed [15], indicators on organization, process and outcome are needed. For quality indicators related to the organization of care, we relied on the chronic care model (CCM). The literature review pointed to the CCM as the most frequently used standard for the organisation of chronic care at the primary care level. It identifies six key health system elements in improving primary care for chronic diseases to be optimized: the healthcare organization, delivery system design, clinical information systems, decision support, self-management support, and community resource linkages [50]. There is growing evidence showing that primary care organisations that implement CCM produces better outcomes for T2D patients [23]. The degree of successful implementation of the CCM is evaluated by the Assessment of Chronic Illness Care tool (ACIC) [51, 52].

Chronic care cannot be captured by a single outcome measure as it entails, by definition, continuous illness management, drawing the attention to both process and outcome indicators. In order to be able to comprehensively measure the chronic disease process and outcomes, a standardized measurement tool of the entire disease continuum thus needs to be developed –one which incorporate all steps of T2D management and outcomes [53, 54]. The document review guided us towards a model known as the cascade of care (CoC). The CoC is a model that outlines the sequential steps in long-term care (screening, diagnosis, linkage with care, in treatment, followed up, and under control). CoC research has helped quantify losses of patients from care (so-called leakages), identify the points of greatest attrition, and target interventions to address these losses [55]. Most studies relying on a CoC approach are on HIV care [55]. However, recently some studies emerged that have shown its relevance for diabetes care. However these are mainly performed outside Europe –in the US [54], India [56], and South and sub-Saharan Africa [54, 57]– which stressed the need for studies in Belgium or other Western European countries which are so far lacking. The CoC was tested among the working group and appreciated as a feasible and useful tool to measure T2D outcomes in Belgium (Flanders) [58].

The integration of both, the ‘CCM’ and the ‘CoC’, within the Structure-Process-Outcome paradigm is presented in Table 1 together with the quality dimensions, corresponding measurable indicators and data level.

### Phase 2: mapping of relevant health data sources

The results of the mapping exercise are summarized in Table 2.

### Phase 3: designing the data framework

#### Selection of data sources

As structure related quality data in primary care practices are not systematically available in Belgium, this data is self-collected at the primary care practice level. For the actual construction of the database, we start therefore with the selection of GP-practices and their patient populations (see phase 4).

A recent Belgian study has shown that for T2D a purely administrative database was the most reliable source to estimate disease prevalence based on dispensed medication in comparison to prescribed or self-reported medication data [59]. Although the quality of registration by GPs shows a positive evolution, recent numbers however indicate that 30% of the patients is still missing in the registrations, and that also among the registered patients, crucial information –especially about clinical parameters– is lacking [60]. Therefore, and in the absence of an exhaustive register of T2D patients or a centralization and standardization of valid data of EHR in Belgium, we have opted for a combination of health insurance and lab data as this provides the necessary longitudinal and most valid and complete information at the patient level. Health insurance data is available through the IMA, a platform where data gathered from the seven Belgian health insurance funds are collected. Medical lab data is until now not centralized, but distributed among more than 60 recognized laboratories active in Belgium.

#### Selection and operationalization of quality measures

The ACIC has been developed to evaluate chronic illness care [51] and was previously validated in Flanders [52, 53]. It is a comprehensive tool targeting generic organization of chronic care across disease populations, and attempts to represent poor to optimal organization and support of care in the CCM areas.

The CoC, which integrates the process and outcome related indicators is operationalized by adapting the bars to the Belgian context and the available IMA and lab data. The different bars can be calculated as percentages in general or per patient group or type of primary care practice. The operationalization of the different bars and on which guidelines or validated sets of quality indicators they are based can be found in Table 3.

**Fig. 2:**
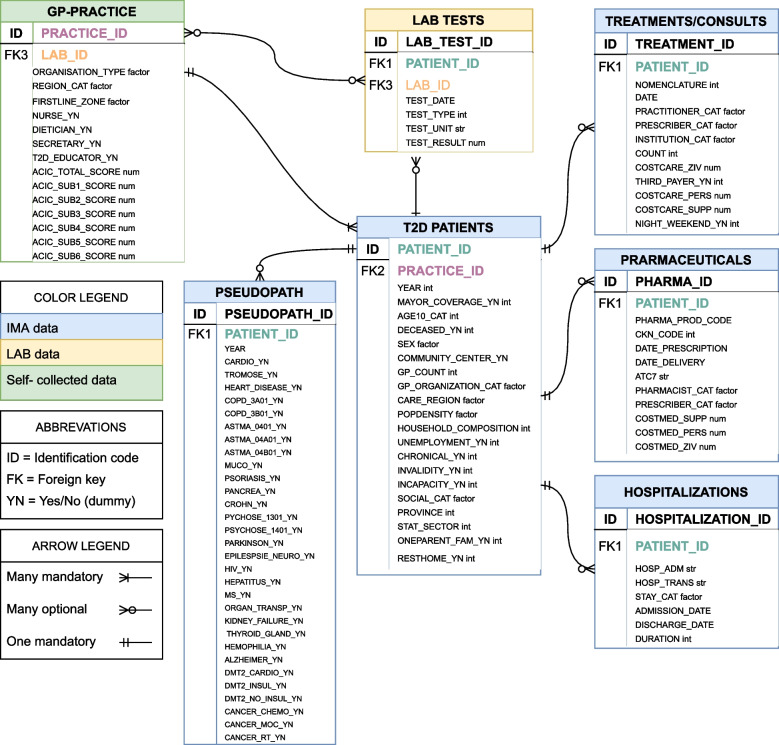
Entity-Relationship Diagram of the Multilevel database of the SCUBY project

In addition, we have constructed an Entity Relationship Diagram (see Fig. 2 and corresponding S4) to give a clear overview of the different *data sources* (IMA, self-collected data and lab data), *entities* (T2D patients, treatments/consults, pharmaceuticals, hospitalizations, lab tests, GP practices), *attributes of the entities* (unique identifier [ID], the foreign key [FK] which make the linkage between the entities possible, and the available variables [e.g. prescriber, count, date, cost]), and *relationships* between them (one to multiple, multiple to one, or one to one; and whether they are potential or mandatory). For all consults, lab tests, treatments, and medicines there is information about when they took place or were delivered (date), by whom (prescriber_cat) and at what cost (cost_ziv, cost_pers, suppl). As a result, we are able to reconstruct the treatment path, including all diabetes relevant consults, lab tests (and results), treatments, and medicines.

### Phase 4: construction of the dataset

#### Selection of GP-practices and patients

The sampling procedure is theoretically driven as it intends to collect a sample with optimal variation of primary care delivery models in order to link structure related quality information to process and outcome indicators. In Belgium (Flanders), primary care facilities can be categorized according to two dimensions, which are also highlighted as relevant health system factors in chronic care research [61]: financing (FFS vs. capitation) and organization (mono- vs. multidisciplinary). A GP-practice is considered as multidisciplinary, when it consists of one or more GPs and at least one nurse and/or dietician. We opted for these two disciplines in addition to the GP, as these play a prominent role in T2D management [62]. Based on these dimensions, four types of GP-practices can be distinguished in theory, but in practice, only three types actually exist in Belgium (Flanders) [63]: (a) monodisciplinary and FFS, (b) multidisciplinary and FFS, and (c) multidisciplinary and a capitation payment system in which patients subscribe for an annual fixed fee. Previous research [63, 64] has already confirmed the relevance of this categorization, showing for example that the control of hemoglobin A1c (HbA1c) and the prescription of statins was better in GP-practices with a fixed capitation system compared to FFS practices [63, 64].

In addition, we captured a range of socioeconomic factors and levels of urbanization, as research [56] has shown the importance of urbanization level in terms of care accessibility. In order to optimise the maximum mix of variability (also in terms of health systems), we opt for Antwerp, Ghent and Kempenland region.

Within each area, the GP-practices are categorized in the three primary care types. As the number of multidisciplinary practices is very low in Flanders, all multidisciplinary practices are selected in the three areas, while for the monodisciplinary practices, a random selection is performed in each area. This has resulted in 66 primary care facilities, which host 277 individual GPs (see S1).

The GPs have a unique identification code and IMA has developed a procedure to identify the patients population of each GP [65]. As patients have also a unique identification code, the linkage between the GP-practices and patient information is possible (see also Fig. 2: ERD and corresponding S4). Within the identified patient populations all T2D patients above 40 years old in year [x-1] are selected. As IMA data lacks diagnostic information, patients diagnosed with T2D are algorithmically identified based on the proxies taking T2D medication (metformin, sulfonylurea, insulin) or having a pre-diabetes pass (registration in this care trajectory allows the reimbursement of a consult with a dietician, podiatrist and diabetes educator) in year [x-1] [48, 49, 66]. This algorithm is validated by medical experts and already used in other research [48, 49, 66]. Individual level data for this patient population for a period of 3 years (from year [x-1] to year [x + 1), with year [x + 1] being the most recent available IMA data).

The total patient size of the 40+ population of the participating GP practices, as identified by IMA, was 85,818 in 2017, of which 7645 (4.2%) were identified as T2D patients. This percentage is somewhat lower than the Belgian percentage of 40+ patients with a self-reported T2D diagnose (8.46%) based on the HIS survey in 2018 [2], probably because our selected population is somewhat younger, based on medication use, and criteria are used to exclude (as good as possible) patients with diabetes type 1.

#### Collecting data at primary care level

The quality of diabetes care is evaluated through structured interviews with observations of GPs and paramedics. Two researchers have scored the practices independently using the ACIC. During a discussion afterwards, a consensus score is defined per item. The six elements of the ACIC are separately assessed by 3 or more items, providing a subscale score: organization of the healthcare delivery system; community linkages; self-management support for patients; decision support for service providers; delivery system redesign; and clinical information system (5 items). The overall ACIC score (an average of the six subscale scores) indicates optimal support for chronic illness.

#### Recruitment of medical labs

During the interviews, the GPs were requested to provide the names of medical laboratories with whom they cooperate. This resulted in a list of seven laboratories active in primary care, which was completed with the labs of the hospitals in the study area, because diabetes patients can also be referred to one of these labs by a specialist or during a hospital stay. This bring us to 12 laboratories which may capture clinical data of the patient population of this study. All these laboratories are recognized for the performance of these medical test by Sciensano and their contact information is publicly available.

We contacted the laboratories telephonically to explain the study, why they are a crucial partners in this project, and what we expect from them. Directly after this first contact, an email was send with more extended information, such as the concrete steps in the collaboration process, which kind of data we request, the format, transmission mode (see S2), and an informed consent which they had to sign if they agreed on the collaboration. The participating labs are connected to the Belgian eHealth platform and use recognized software, formats and standardized codes (the national set of LOINC codes)(see S2). This enables us to perform a valid harmonization of the data of the different labs.

#### Access and application procedures

Three types of approval were needed for the construction of the database including IMA data and its linkage to the lab and self-collected data at the GP-practices: (1) approval from the relevant ethical commission; (2) internal approval from the database administrator organization and (3) approval from the information protection commission [67].

1. We applied for the Ethics committee of Antwerp University Hospital, which advises on all ethical aspects in the context of scientific research.
2. To obtain data and collaboration of the IMA a declaration of interest needed to be set up between researchers of the project and the IMA programme managers. The research proposal was discussed and after they agreed on collaboration, the project was in short presented to IMA registry directory board for approval. After this internal approval, a final detailed selection of the data and variables with the required motivation was prepared with the support of data managers of IMA.
3. Thereafter approval was requested of the institution of social security and health, a subcommittee of the Information Protection Committee. The full process from application to approval can take 6 months and during this process additional information about the linkage procedure can be requested or a small cell risk analysis to ensure privacy of the included individuals.

#### Data trajectory and linkage procedure

The data trajectory is developed in collaboration with IMA and has been submitted to eHealth in advance of the application for approval of the ethical commission and the information protection commission. A common unique identifier (i.e. social security number = INSZ or RIZIV) made deterministic linking possible. For privacy reasons, two trusted Third Parties (TTPs) ‘eHealth’ (a data platform of the federal government) and ‘Crossroads Bank for Social Security’ (CBSS) are responsible for this deterministic matching procedure using multiple encrypted social security numbers.

The linkage procedure consists of 13 steps (see flow chart and steps in S3) of data coding or decoding and data transfers (using the principle of random transport number ‘RN’) needed to ensure that none of the involved parties would have access to the sensitive data and the social security numbers. Only researchers of the project have access to the complete linked database without unique identifiers using a virtual private network (VPN).

As a result of this procedure, for 73.8% (or 5643 patients) of the 7645 T2D patients identified by IMA, the linkage with medical lab data was possible. For 26.2% of the patients this linkage was not possible, because only 8 of the 12 labs participated. (The most common reason of not participating was the high work load during the COVID-19 period.) For these latter patients however, we have information about which lab tests have been done (when, the cost, etc.), by linking the population data to the ‘treatments/consults’ data (see Fig. 2: ERD).

### Phase 5: Formulating research questions and building the corresponding analysis models for the dataset

**Fig. 3:**
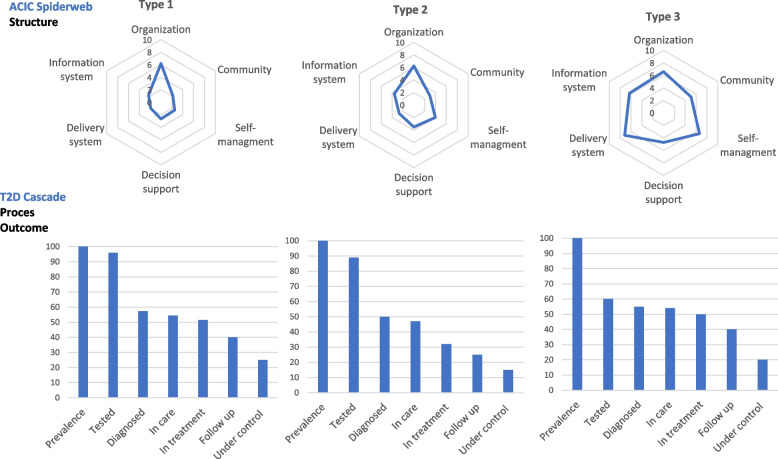
Fictive visualization of the quality indicators of integrated T2D care stratified by primary care practice type

The data will provide the opportunity to develop evaluation questions about the three dimensions of Donabedian’s quality framework and the correlation between the dimensions structure, process and outcomes. To assess and visualize the QIs in a comprehensive way, two types of graphs will be constructed: the organization related indicators measured by the ACIC will be presented in a spiderweb [68] and the process and outcome indicators in a bar chart visualizing the CoC [55]. The spiderwebs linked to the CoCs can be stratified by health systems and/or patients groups to describe quality differences between the three GP-practice types and/or vulnerable and non-vulnerable patients (see a fictive example in Fig. 3).

To further analyse which factors (e.g. socioeconomic status of the patient, gender, age) are related to the drops in the cascade, the drops will be used as dichotomous outcome variables of bivariate and multivariable logistic regression analyses. Knowledge on these leakages will inform healthcare reforms targeting those T2D patients currently not tested, undiagnosed, unlinked to care, not taking treatment, poor followed up, and not achieving glycemic control.

By combining the patient level data with the GP-practice data, we will be able to explain observed differences in process and outcome related quality measures between GP-practices by the degree of successful implementation of the CCM. The multilevel structure of the data –patients (level 1) clustered within GP-practices (level 2) (see figure in S5)– will allow for multilevel analyses to estimate the impact of structure related factors (GP-practice level) on patients’ probabilities of reaching (or not reaching) a particular stage in the cascade.

## Discussion and conclusion

This study has developed a unique multilevel data framework for assessing the quality of integrated T2D care and operationalized this framework in the fragmented Belgium healthcare and data landscape. The integration of (1) the Structure-Process-Outcome model of Donabedian with (2) a CoC approach enables us to not only assess the quality of care via the different dimensions, but to also investigate and understand differences in the quality of care and the impact of organization related factors on the total care continuum instead of only patient outcomes [69]. The clustering of patients in primary care practices and the availability of data at both levels enables us to relate differences in the quality of care between patient groups and types of GP-practices to organizational factors of the primary care practices and the extent of the implementation of the CCM.

Another opportunity of the developed database is that patients can be followed retrospectively over 3 years, which renders the study of more advanced indicators such as stepped-care indicators and clinical action oriented quality indicators possible enables on top of the classical, rather ‘rough’, quality indicators [68, 70]. These advanced indicators look at the clinical path and require longitudinal data to also look at the actions (initiation and/or intensification of treatment) that are taken by healthcare providers (process) after certain outcome values (with regard to eGFR, HbA1c, albuminuria and/or LDL-c) in the patient [71–73]. Until now, this has only been done in Belgium among diabetes patients treated with at least two insulin injections per day [47]. Research has shown the added value of these indicators, namely a better predictive value for hard health outcomes (such as micro and/or macro vascular complications) [47], as they depend less on patient characteristics.

The operationalization of this measurement framework of quality of care in a fragmented health data landscape as Belgium has revealed several problems and challenges. Due to the lack of a diabetes register and poor coordination between the primary, secondary and tertiary care level, quality of care research is often limited to datasets with full information of only one level of care and/or only covering one part of the patients (e.g. the most severe). For example, studies using EHR data [39, 62] do often not fully capture specialist and inpatient care, and research [74] relying on data of hospitals (specialist and inpatient care) do not cover less severe patients, mainly treated at primary care level. Moreover, the majority of studies are only addressing one or two dimensions of care, because studying the three quality dimensions in a comprehensive way, requires different types of data (e.g. medication use and/or prescription data, health care use data, clinical data) and data at different levels (e.g. the organization, health provider and patient level), which are rarely included in one linked dataset. For example, quality of care research using health insurance data predominantly focusses on the process dimension, because of a lack of clinical information [74]. In studies relying only on EHRs, survey data, or health claims data organizational characteristics and the structure related quality indicators are rarely related to physician process and patients outcomes in studies [73],

With this study we aim to provide solutions to these challenges. The unique and comprehensive multilevel dataset for the assessment and study of integrated T2D care has several strengths. As it consists of health insurance data, it includes in addition to basic sociodemographic information, extensive health care and medication data, cost information and professional information of the registered health care provider (e.g. financing system, organization type ‘group vs. solo’). The data is longitudinal, exhaustive and very reliable, as the quality is not depending on the quality of the registration or reporting by the health care providers or patients. This because registration is routinely, standardized and necessary for reimbursement. One of the main limitations of health insurance data, namely the lack of clinical data, has been met in our dataset by the linkage with lab data. The linkage with self-collected primary care level data about the degree of implementation of the dimensions of the CCM makes this dataset very unique, including information about the three dimensions of quality of care. As a result, the data framework is adequate for quality assessment as well as for multilevel research to explain quality differences among patient groups and primary care organizations, through patient and GP-practice characteristics.

### Limitations and future research

The main limitation of this project is to be found within its complexity. Linking the databases, which is a crucial step to measure the whole care continuum is complicated and requires much time and manpower. However, measuring quality is only helpful if actions can be taken and measurements can be repeated. If this is desired, the whole process needs to be redone from the start, inclusively the application at the different boards and the collection of the ACIC data. This study is therefore also a strong plea for linking lab and health insurance data and creating a routine diabetes register.

A comprehensive assessment of quality of care from a triple aim outcome perspective also includes patient-centered outcomes and experiences and the cost of care [75]. Although the health insurance data provide the opportunity to make cost-effectiveness analyses, given detailed cost-information is available (See Fig. 2: ERD), the analysis were beyond the scope of this paper. The most important Patient-centered outcomes for diabetes have been developed by the International Consortium for Health Outcomes Measurement (ICHOM) (including the WO-5, PHQ9 and PAID questionnaire) [76] and the patient perspectives of healthcare in first line are currently being developed and tested in a OECD project ‘Patient-reported Indicator Surveys (PaRIS)’ [31, 71, 72]. The use of this PREM and PROM set and the ICHOM set of Patient-Centred Outcome Measures for T2D was not yet possible in the current study. However, in the future – the negotiations are already started together with KUL, Sciensano, Vivel, IMA, Pharmaflux– we hope that there are possibilities to measure these patient reported indicators systematically and link them to the integrated data.

## Conclusion

The phased approach of the development of a theoretical framework of quality of care, and its translation in a data framework with measurable quality indicators, can be used as a template for the assessment of the quality of diabetes care in other countries with a complex data landscape and for other chronic diseases such as cardiovascular diseases, hypertension, COPD, etc..

The use of administrative health data and the linkage of different data sources is very enriching for quality of care research, but remains challenging and it requires a strong collaboration in different domains, such as expertise in the clinical field, statistics, epidemiology, and data management, as well as between academics and database administrators and privacy commission bodies.

## Supplementary Information

**Additional file 1.**

**Additional file 2.**

**Additional file 3.**

**Additional file 4.**

**Additional file 5.**

## Data Availability

Not applicable. No dataset was used in this research article. All database administrators can be contacted freely.

## Abbreviations

T2D: Type 2 Diabetes
HIS: Health Information System
QI: Quality Indicators
EHR: Electronic Health Record
GP: General Practitioner
FFS: Fee-For-Service
CCM: Chronic Care Model
ACIC: Assessment of Chronic Illness Care tool
CoC: Cascade of Care
IMA: Intermutualistic Agency
HbA1c: Hemoglobin A1c
ERD: Entity Relationship Diagram
FK: Foreign Key
ID: Identification code
Loinc: Logical Observation Identifiers Names and Code
TTP: Trusted Third Party
CBSS: Crossroads Bank for Social Security
RN: Random transport Number
VPN: Virtual Private Network
eGFR: estimated Glomerular Filtration Rate
LDL-c: Low-Density Lipoprotein cholesterol
OECD: Organisation for Economic Co-operation and Development
PaRIS: Patient-Reported Indicator Survey
PROM: Patient-Reported Outcome Measure
PREM: Patient-Reported Experience Measure
ICHOM: International Consortium for Health Outcomes Measurement
WHO-5: World Health Organisation- Five Well-Being Index (WHO-5)
PHQ9: Patient Health Questionnaire-9
PAID: Problem Areas In Diabetes Scale
COPD: Chronic obstructive pulmonary disease

## References

[1] World Health Organization. Global report on diabetes. World Health Organization. 2016. https://apps.who.int/iris/handle/10665/204871WHO Accessed 23 Mar 2021.

[2] HIS-BELHES. Health interview survey - Belgian health examination survey*.* Sciensano. 2018. https://www.sciensano.be/en/projects/health-examination-survey Accessed 23 Mar 2021.

[3] International diabetes federation. Recommendations for managing type 2 diabetes in primary care. 2017. http://www.idf.org/managing-type2-diabetes Accessed 7 July 2022.

[4] Meneghini L, American Diabetes Association (2020). Medical Management of Type 2 diabetes.

[5] Scottish Intercollegiate Guidelines Network (SIGN). Pharmacological management of glycaemic control in people with type 2 diabetes. Edinburgh: SIGN. 2017. (SIGN publication no. 154). http://www.sign.ac.uk Accessed 7 July 2022.

[6] Koeck P, et al. Guidelines for good medical practice: Diabetes type 2 [Richtlijn voor goede medische praktijkvoering: Diabetes melitus type 2]. Domus Medica. 2015. https://www.domusmedica.be/sites/default/files/Richtlijn%20Diabetes%20%28correctie%2019-01-12%29.pdf Accessed 7 July 2022.

[7] Chronic Disease Control (CDC). National diabetes prevention program. 2021. https://www.cdc.gov/diabetes/prevention/index.html Accessed 7 July 2022.

[8] National Institute for health and clinical excellence National Collaborating Centre for chronic conditions. Type 2 diabetes: national clinical guideline for management in primary and secondary care (update). London: Royal College of Physicians; 2008. 21678628

[9] Nicolucci A, Greenfield S, Mattke S (2006). Selecting indicators for the quality of diabetes care at the health systems level in OECD countries. Int J Qual Health Care.

[10] Felton A, Hall M (2015). Diabetes in Europe policy puzzle: the state we are in. Int Diab Nurs.

[11] Kahn R (2010). Age at initiation and frequency of screening to detect type 2 diabetes: a cost-effectiveness analysis. Lancet..

[12] Gilson L, Doherty JE, Loewenson R, et al. Challenging inequity through health systems: final report knowledge network on health systems. Who commission on the social determinants of health. World Health Organization, Geneva, 2007. http://www.who.int/social_determinants/resources/csdh_media/hskn_final_2007_en.pdf Accessed 22 Feb 2021.

[13] Grp TS (2010). Health systems, patients factors, and quality of Care for Diabetes a synthesis of findings from the TRIAD study. Diabetes Care.

[14] Fokkens AS, Wiegersma PA, van der Meer K, et al. Structured diabetes care leads to differences in organization of care in general practices: the healthcare professional and patient perspective. *Bmc*. Health Serv Res. 2011;11(113). 10.1186/1472-6963-11-113. 10.1186/1472-6963-11-113 PMC3116472 21600064

[15] Donabedian A (1988). The quality of care - how can it be assessed. Jama-J Am Med Assoc.

[16] Donabedian A (1985). The epidemiology of quality. Inquiry.

[17] Donabedian A (1985). 20 years of research on the quality of medical-care - 1964-1984. Eval Health Professions.

[18] Donabedian A (1984). Quality, cost, and cost containment. Nurs Outlook.

[19] Buffel V, Nicaise I. ESPN thematic report on inequalities in access to healthcare: Belgium. Europan Social Policy Network 2018 European Commission Brussels. 2019:1–21.

[20] Goderis G (2009). Type 2 diabetes in primary Care in Belgium: need for structured shared care. Exp Clin Endocrinol Diabetes.

[21] Vrijhoef HJM (2002). The nurse specialist as main care-provider for patients with type 2 diabetes in a primary care setting: effects on patient outcomes. Int J Nurs Stud.

[22] Shojania KG, Ranji SR, Shaw LK, et al. Closing the Quality Gap: A Critical Analysis of Quality Improvement Strategies (Vol. 2: Diabetes Care). Rockville (MD): Agency for Healthcare Research and Quality (US). 2004 (Technical Reviews, No. 9.2.) https://www.ncbi.nlm.nih.gov/books/NBK43938/ Accessed 23 Feb 2021. 20734526

[23] Stellefson M, Dipnarine K, Stopka C. The chronic care model and diabetes management in US primary care settings: a systematic review. Prev Chronic Dis 2013;10(e25). 10.5888/pcd10.120180. 10.5888/pcd10.120180 PMC3604796 23428085

[24] Mahdavi M (2019). The relationship between context, structure, and processes with outcomes of 6 regional diabetes networks in Europe. PLoS One.

[25] Collins MM (2009). Quality of life and quality of Care in Patients with Diabetes Experiencing Different Models of care. Diabetes Care.

[26] World Health Organization (Europe) and international diabetes federation (Europe). Diabetes care and research in Europe: the Saint Vincent declaration. Diabet Med 1990; 7(4): 360. 2140091

[27] Piwernetz K (2001). DIABCARE quality network in Europe-a model for quality management in chronic diseases. Int Clin Psychopharmacol.

[28] de Beaufort CE (2003). European Union diabetes indicators: fact or fiction?. Eur J Pub Health.

[29] Final Report of the EUCID Project, 2005. http://ec.europa.eu/health/ph_projects/2005/action1/docs/action1_2005_frep_11_en.pdf Accessed 7 July 2022.

[30] Cunningham SG (2016). Core standards of the EUBIROD project. Defining a European diabetes data dictionary for clinical audit and healthcare delivery. Methods Inf Med.

[31] Scarpetta S, et al. The PaRIS initiative: helping healthcare policies to do better for patients. OECD. 2018; 10.1787/51134dc8-en. Accessed 7 July 2022.

[32] Nocella JM (2016). Structure, process, and outcomes of care in a telemonitoring program for patients with type 2 diabetes. Patient Relat Outcome Meas.

[33] Bak JCG (2021). National diabetes registries: do they make a difference?. Acta Diabetol.

[34] Peterson A (2015). Collaboratively improving diabetes Care in Sweden Using a National Quality Register: successes and challenges-a case study. Qual Manag Health Care.

[35] Eliasson B, Gudbjörnsdottir S (2014). Diabetes care-improvement through measurement. Diabetes Res Clin Pract.

[36] Sigfrid LA (2006). Using the UK primary care quality and outcomes framework to audit health care equity: preliminary data on diabetes management. J Public Health.

[37] Jorgensen ME (2016). The Danish adult diabetes registry. Clin Epidemiol.

[38] Gnavi R (2009). Determinants of quality in diabetes care process the population-based Torino study. Diabetes Care.

[39] Truyers C, et al. The Intego database: background, methods and basic results of a Flemish general practice-based continuous morbidity registration project. Bmc Med Informatics Decision Making. 2014;14(48). 10.1186/1472-6947-14-48. 10.1186/1472-6947-14-48 PMC4067630 24906941

[40] Delvaux N (2018). Health data for research through a Nationwide privacy-proof system in Belgium: design and implementation. JMIR Med Inform.

[41] van Olmen J, et al. Scale-up integrated care for diabetes and hypertension in Cambodia, Slovenia and Belgium (SCUBY): a study design for a quasi-experimental multiple case study. Glob Health Action. 2020;13(1). 10.1080/16549716.2020.1824382. 10.1080/16549716.2020.1824382 PMC7594757 33373278

[42] RIZIV. The conditions for the integrated practice premium for general practice medicine. 2017. https://www.inami.fgov.be/nl/professionals/individuelezorgverleners/artsen/hulp/geintegreerde-praktijk/Paginas/toelichting-geïntegreerde-praktijkpremie.aspx#U_gebruikt_E-diensten_en_bereikt_de_drempel_voor_een_aantal_parameters Accessed 10 Mar 2021.

[43] Federal government, e-Health platform. 2021. https://www.ehealth.fgov.be/ehealthplatform/nl Accessed 7 July 2022.

[44] Federal goverment, Mijn Gezondheid [My Health]. 2020. https://www.mijngezondheid.belgie.be. Accessed 7 July 2022.

[45] Carinci F, et al. Making use of comparable health data to improve quality of care and outcomes in diabetes: the EUBIROD review of diabetes registries and data sources in Europe. Frontiers in Clinical Diabetes and Healthcare. 2021:2. 10.3389/fcdhc.2021.744516. 10.3389/fcdhc.2021.744516 PMC10012140 36994337

[46] Debacker N (2008). Organization of a quality-assurance project in all Belgian multidisciplinary diabetes centres treating insulin-treated diabetes patients: 5 years’ experience. Diabet Med J Br Diabet Assoc.

[47] Lavens A, Doggen K, Mathieu C, et al. Clinical action measures improve the reliability of feedback on quality of care in diabetes centres: a retrospective cohort study. BMC Health Serv Res. 2016;16(424). 10.1186/s12913-016-1670-5. 10.1186/s12913-016-1670-5 PMC4995611 27553193

[48] Van Casteren V, Bossuyt N, Moreels S, et al. De zorgtrajecten diabetes mellitus type 2 en chronische nierinsufficiëntie: impact op de kwaliteit van zorg, 2013. Brussel: Wetenschappelijk Instituut Volksgezondheid (WIV-ISP). Brussels, Belgium, 2013: D/2013/2505/24.

[49] Sunaert P, Bastiaens H, Nobels F, et al. Effectiveness of the introduction of a chronic care model-based program for type 2 diabetes in Belgium. BMC Health Serv Res. 2010;10(207). 10.1186/1472-6963-10-207. 10.1186/1472-6963-10-207 PMC2912901 20630062

[50] Baptista DR, et al. The chronic care model for type 2 diabetes: a systematic review. Diabetol Metabolic Syndrome. 2016;8(7). 10.1186/s13098-015-0119-z. 10.1186/s13098-015-0119-z PMC4722715 26807158

[51] Bonomi AE (2002). Assessment of chronic illness care (ACIC): a practical tool to measure quality improvement. Health Serv Res.

[52] Cramm JM, et al. Development and validation of a short version of the assessment of chronic illness care (ACIC) in Dutch disease management programs. Health Qual Life Outcomes. 2011;9(49). 10.1186/1477-7525-9-49. 10.1186/1477-7525-9-49 PMC3141373 21726439

[53] Ali MK (2014). A Cascade of Care for Diabetes in the United States: visualizing the gaps. Ann Intern Med.

[54] Stokes A, et al. Prevalence and unmet need for diabetes care across the care continuum in a national sample of south African adults: evidence from the SANHANES-1, 2011-2012. PLoS One 2017;12(10). 10.1371/journal.pone.0184264. 10.1371/journal.pone.0184264 PMC5624573 28968435

[55] Haber N (2016). Constructing the cascade of HIV care: methods for measurement. Curr Opin HIV AIDS.

[56] Prenissl J (2019). Variation in health system performance for managing diabetes among states in India: a cross-sectional study of individuals aged 15 to 49 years. BMC Med.

[57] Price AJ (2018). Prevalence of obesity, hypertension, and diabetes, and cascade of care in sub-Saharan Africa: a cross-sectional, population-based study in rural and urban Malawi. Lancet Diabetes Endocrinol.

[58] Danhieux K (2020). Waar haken mensen in de zorg voor diabetes en hypertensie af? Voorstelling van het SCale-Up diaBetes and hYpertensive care (SCUBY)-project. Huisarts Nu.

[59] Vaes B (2018). Estimating the prevalence of diabetes mellitus and thyroid disorders using medication data in Flanders, Belgium. Eur J Public Health.

[60] Goosens M, et al. De zorgtrajecten diabetes mellitus type 2 en chronische nierinsufficiëntie en kwaliteit van zorg: EVACQ Evaluation of Ambulatory Care Quality 2017–2019. Sciensano: Brussels, 2019: D/2019/14.440/85.

[61] Kiran T (2014). The relationship between primary care models and processes of diabetes Care in Ontario. Can J Diabetes.

[62] Van Casteren V, et al. Does the Belgian diabetes type 2 care trajectory improve quality of care for diabetes patients? Arch Public Health. 2017;73(31). 10.1186/s13690-015-0080-1. 10.1186/s13690-015-0080-1 PMC4499949 26171143

[63] Boutsen M, et al. Vergelijking van kost en kwaliteit van twee financieringssystemen voor de eerstelijnszorg in België: een update. Intermutualistisch Agentschap – Agence Intermutualiste*.* 2017. https://ima-aim.be/IMG/pdf/maisons_medicales_ima-2.pdf Accessed 22 Jan 2021.

[64] KCE Reports 85A. Vergelijking van kost en kwaliteit van twee financieringssystemen voor de eerstelijnszorg in België. 2008. https://kce.fgov.be/report/85A Accessed 30 Jan 2021.

[65] Intermutualistisch Agentschap (IMA), Patiëntenbestanden en contacten van huisartsen: overzicht methodologie en lay-out resultaten, in PROJECT IMA-CHTA [IMA2011001]. 2018. https://www.ima-aim.be/IMG/pdf/methodologie_patientenbestand_huisartsen_externen-2021 Accessed 3 Jan 2021.

[66] Claesen M, Gillard P, De Smet F (2016). Mortality in individuals treated with glucose-lowering agents: a large, controlled cohort study. J Clin Endocrinol Metab.

[67] Maetens A, De Schreye R, Faes K (2016). Using linked administrative and disease-specific databases to study end-of-life care on a population level. Bmc Palliative Care.

[68] Sidorenkov G, van Boven JFM, Hoekstra T (2018). HbA1c response after insulin initiation in patients with type 2 diabetes mellitus in real life practice: identifying distinct subgroups. Diabetes Obes Metab.

[69] Yeoh EK, Wong MCS, Wong ELY (2018). Benefits and limitations of implementing chronic care model (CCM) in primary care programs: a systematic review. Int J Cardiol.

[70] Sidorenkov G, Voorham J, de Zeeuw D (2013). Treatment quality indicators predict short-term outcomes in patients with diabetes: a prospective cohort study using the GIANTT database. BMJ Quality Safety.

[71] OECD. Patient-reported Indicator Surveys (PaRIS). 2020. http://www.oecd.org/health/paris/ Accessed 10 Feb 2021.

[72] Desomer A, et al. Het gebruik van patiëntuitkomsten en -ervaringen (PROMs/PREMs) voor klinische en beleidsdoeleinden. Health Services Research: KCE Reports 303A, 2018.

[73] Devos C, Lefèvre M, Obyn C, et al. Performance of the Belgian health system – report 2019. Health Services Research (HSR) Brussels: Belgian Health Care Knowledge Centre (KCE), 2019. KCE Reports 313(D/2019/10.273/34).

[74] Tanaka H, Tomio J, Sugiyama T, Kobayashi Y (2016). Process quality of diabetes care under favorable access to healthcare: a 2-year longitudinal study using claims data in Japan. BMJ Open Diabetes Res Care.

[75] Hanefeld J, Powell-Jackson T, Balabanova D (2017). Understanding and measuring quality of care: dealing with complexity. Bull World Health Organ.

[76] ICHOM Diabetes in Adults Working Group, Type 1 and Type 2 Diabetes in Adults.2018. www.ichom.org/medical-conditions/diabetes Accessed 7 July 2022.

