# Correction: Development and operationalization of a data framework to assess quality of integrated diabetes care in the fragmented data landscape of Belgium

**DOI:** 10.1186/s12913-022-08965-5

**Published:** 2022-12-19

**Authors:** Veerle Buffel, Katrien Danhieux, Philippe Bos, Roy Remmen, Josefien Van Olmen, Edwin Wouters

**Affiliations:** 1 Department of Sociology, University of Antwerp, Antwerp, Belgium; 2 Department of Family Medicine and Population Health, University of Antwerp, Antwerp, Belgium

**Correction: BMC Health Services Research (2022) 22:1257**

**https://doi.org/10.1186/s12913-022-08625-8**

Following publication of the original article [1], the authors identified an error in the author names. The given names and family names of all authors (except for one) were erroneously transposed.

The incorrect author names are: Buffel Veerle, Danhieux Katrien, Remmen Roy, Van Olmen Josefien and Wouters Edwin

The correct author names are: Veerle Buffel, Katrien Danhieux, Roy Remmen, Josefien Van Olmen and Edwin Wouters

The author group has been updated above and the original article [1] has been corrected.
