# Supplementary material for: Development and operationalization of a data framework to assess quality of integrated diabetes care in the fragmented data landscape of Belgium
Source: BMC Health Serv Res. 2022 Oct 18;22:1257. doi: 10.1186/s12913-022-08625-8 (PMC9578257; doi:10.1186/s12913-022-08625-8)
Supplement: Supplementary file 1 — Additional file 1. [file 12913_2022_8625_MOESM1_ESM.docx]

Viii. ADDITIONAL FILES

| *Additional file 1. Table: Number and type of participating GP-practices by study area* | | | | |
| --- | --- | --- | --- | --- |
|  |  | **Study area** | | |
|  | **Region** | Antwerp | Ghent | Kempen |
|  | **Rural/Urban** | urban | urban | rural |
| **Type of primary care**  **practice** | Monodisciplinary + Fee-for-service | 10 | 10 | 10 |
|  | Multidisciplinary + Fee-for-service | 9 | 4 | 6 |
|  | Multidisciplinary + capitation system | 7 | 9 | 1 |
