# Supplementary material for: Development and operationalization of a data framework to assess quality of integrated diabetes care in the fragmented data landscape of Belgium
Source: BMC Health Serv Res. 2022 Oct 18;22:1257. doi: 10.1186/s12913-022-08625-8 (PMC9578257; doi:10.1186/s12913-022-08625-8)
Supplement: Supplementary file 2 — Additional file 2. [file 12913_2022_8625_MOESM2_ESM.docx]

*Additional file 2. Table: Requested information of the medical labs*

| **CONTENT OF LAB DATA** | | | |
| --- | --- | --- | --- |
| **ZONE** | **DESCRIPTION** | | |
| PATIENT_ID | Unique identifier of patient | | |
| LAB_ID | Unique identifier of lab | | |
| TEST_DATE | Date of test | | |
| TEST_TYPE | **Type of test** | **Unit** | **Notes** |
|  | Glycemia | Mg/dl | glucose sober AND not sober |
|  | Hba1c | Mmol/mol  and % |  |
|  | Micro albumineria | Mg/L | on portion AND on 24 hours of urine |
|  | Macro albumineria | Mg/L | on portion AND on 24 hours of urine |
|  | Normo albuminuria | Mg/L | on portion AND on 24 hours of urine |
|  | Cholesterol total | mg/dl | Measured AND calculated |
|  | HDL-cholesterol | mg/dl | Measured AND calculated |
|  | LDL-cholesterol | mg/dl | Measured AND calculated |
|  | Triglycerides | mg/dl | Measured AND calculated |
|  | eGFR | MI/minute 1,7m2 | MDRD AND 24h urine |
| TEST_RESULT | Result of the test | | |
| TEST_UNIT | Unit of the result of the test | | |
| **FORMAT AND TRANSFER MODE** | | | |
| FORMAT | Long format: each row of the dataset is a unique lab test (so several rows per patient are possible)  File type: The data may be delivered in an excel, csv or SAS file | | |
| TRANSFER MODE | Gpg4Win is used as file and email encryption software, allowing users to secure emails and transport files using encryption and digital signatures.  The software uses a key pair: a public key for encryption and a private key for decryption. | | |
