# Supplementary material for: Development and operationalization of a data framework to assess quality of integrated diabetes care in the fragmented data landscape of Belgium
Source: BMC Health Serv Res. 2022 Oct 18;22:1257. doi: 10.1186/s12913-022-08625-8 (PMC9578257; doi:10.1186/s12913-022-08625-8)

*Additional file 3. Flow chart and steps: The linkage procedure of data coding or decoding and data transfers*

The data-applicant – the researchers of the university of Antwerp in this project – provided the participation list with RIZIV numbers (gp_id) of the m general practitioners (gp_id[1] – gp_id[m]) and the n associated medical labs (lab_id[1] – lab_id[n]). One GP can be associated with more than one lab and one lab can also work together with more than one GP, for example (m: the number of participating GPs; n: the number of participating medical labs)

Lab_id[1] gp_id[1]

Lab_id[1] gp_id[2]

… …

Lab_id[1] gp_id[m]

Lab_id[2] gp_id[1]

Lab_id[2] gp_id[4]

… …

Lab_id[n] gp_id[m]

1. The participation list gp_id[m]/lab_id[n] is transferred by the applicant to the IMA.
2. Based on the participation list gd_id[m]/lab_id[n] and the other inclusion criteria specified by the applicant, the IMA creates a list of all unique C2 (=coding) (distinct(C2)) for this project and selects also the necessary IMA data on C2 (C2/IMA_data):

(2.1) IMA transfers the list distinct(C2) to the TTP VI (CBSS).

(2.2) IMA transfers the C2/IMA_data to the TTP VI (CBSS). Note: this step can also be done later, but it has to be done at least when step 11 is finished.

1. The TTP VI (CBSS) converts each C2 from the list distinct(C2) to a unique C1 (=coding) and transfers the new list distinct (C1) to the SPOC NIC (= Security officer of the National InterMutualistic College)
2. The SPOC NIC converts each C1 from the list distinct(C1) to a unique INSZ (= social security number) and transfers the new list distinct(INSZ) to the TTP eHealth. The TTP eHealth converts each INSZ based on the list distinct(INSZ) to a unique Cproject. The TTP ehealth creates for each INSZ also a RN (= random transport number), and based on this, the TTP ehealth creates two new lists distinct(INSZ/RN) and distinct(RN/Cproject):
3. 5.

(5.1) TTP eHealth transfers the list distinct(INSZ/RN) to the SPOC NIC.

(5.2) TTP eHealth transfers the list distinct(RN/Cproject) to the TTP VI(CBSS).

1. the SPOC NIC converts each INSZ from the list distinct(INSZ/RN) to a unique C1 and send the new list distinct(C1/RN) to the TTP VI (CBSS). IMA composes on the basis of the participation list of gp_id[m]/lab_id[n] from step 1 n lists of patients C2[m]/lab_id[n], with all unique patients brought to C2 from each GP who can be associated with a specific lab. Example of patient list 1 (gp_id will not be included):

Lab_id[1] C2[1] (of gp_id[1])

Lab_id[1] C2[2] (of gp_id[1])

Lab_id[1] C2[3] (of gp_id[2])

Lab_id[1] C2[4] (of gp_id[2] and of gp_id[1])

… …

Lab_id[1] C2[x] (of gp_id[m])

1. IMA transfer the n x C2[m]/lab_id[n] patient lists to the TTP VI (CBSS).
2. TTP VI (CBSS) converts each C2 from each patient list C2[m]/lab_id[n] to the unique C1 and send the n new lists C1[m]/lab_id[n] to the SPOC NIC.
3. SPOC NIC converts each C1 from each patient list C1[m]/lab_id[n] to the unique INSZ and send the new lists INSZ[m]/lab_id[n] to TTP eHealth.
4. TTP ehealth converts each INSZ from the n patient lists INSZ[m]/lab_id[n] to the unique RN and send each new list INSZ[m]/RN[n] to the correct lab[n] based on the lab_id.
5. Each lab send the requested study-data on RN (RN[n]/labdata[n]) to the TTP VI(CBSS).
6. TTP VI (CBSS) converts each RN from the n datasets RN[n]/lab­­_data[n]) to the unique Cproject, and converts also each C2 from the IMA data (see step 2.2) to the unique Cproject. Thereafter, TTP VI (CBSS) places all data – brought on the Cproject – in the IMA Project Datawarehouse.
7. The n datasets CProject[n]/lab_data[n] and all datasets Cproject/IMA_data are made available to the researchers


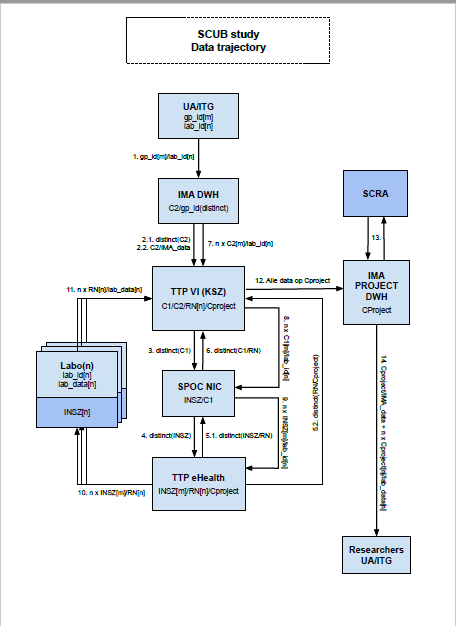

Supplement: Supplementary file 3 — Additional file 3. [file 12913_2022_8625_MOESM3_ESM.docx]
