# Supplementary material for: Development and operationalization of a data framework to assess quality of integrated diabetes care in the fragmented data landscape of Belgium
Source: BMC Health Serv Res. 2022 Oct 18;22:1257. doi: 10.1186/s12913-022-08625-8 (PMC9578257; doi:10.1186/s12913-022-08625-8)
Supplement: Supplementary file 4 — Additional file 4. [file 12913_2022_8625_MOESM4_ESM.docx]

*Additional file 4. Table: Corresponding table of the Entity Relationship Diagram (ERD: Figure 2) including descriptions of the data entities and variables*

| **ENTITY** | **VARIABLE** | **DESCRIPTION** |
| --- | --- | --- |
| GP PRACTICE |  | Self-collected data in the participating GP-practices |
|  | PATIENT_ID | Unique identifier of patient |
|  | LAB_ID | Unique identifier of lab |
|  | ORGANISATION_TYPE | Solo and fee-for-service, monodisciplinary and fee-for-service, multidisciplinary and capitation system |
|  | REGION_CAT | Region of the GP practice: Ghent, Antwerp, Kempen |
|  | FIRSTLINE_ZONE | Primary care zone of the GP practice |
|  | NURSE_YN | Whether there is a nurse in the GP practice: yes/no |
|  | DIETICIAN_YN | Whether there is a dietician in the GP practice: yes/no |
|  | SECRETARY_YN | Whether there is a secretary in the GP practice: yes/no |
|  | T2D_EDUCATOR_YN | Where is a T2D educator in the GP practice: yes/no |
|  | TOTAL_ACIC_SCORE | The total score of the GP practice on the assessment of chronic illness care tool |
|  | ACIC_SUB1_SCORE to ACIC_SUB6_SCORE | The scores of the GP practice on the subdimensions of the assessment of chronic illness care tool (1 ‘Organization’, 2 ‘Community linkages’, 3 ‘Self-management support’, 4 ‘Decision support’, 5 ‘Delivery system design’ 6 ‘Information systems’ |
| LAB_TESTS |  | Data delivered by the participating labs |
|  | LAB_TEST_ID | Unique identifier of lab test |
|  | PATIENT_ID | Unique identifier of patient |
|  | LAB_ID | Unique identifier of lab |
|  | TEST_DATE | Date of the lab test |
|  | TEST_TYPE | Type of lab test: Glycemia, Hba1c, Micro albumineria, Macro albumineria, Normo albuminuria, Cholesterol total, HDL-cholesterol, LDL-cholesterol, Triglycerides, or eGFR |
|  | TEST_UNIT | Measurement unit of the result of the test: Mg/dl, Mg/L, Mmol/mol, %, MI/minute or 1,7m2 |
|  | TEST_RESULT | The result of the lab test |
| T2D_PATIENTS |  | Data delivered by the IMA population dataset |
|  | PATIENT_ID | Unique identifier of patient |
|  | PRACTICE_ID | Unique identifier of GP-Practice of the patient (based on the IMA procedure to identify the patients population of each GP) |
|  | YEAR | 2017, 2018 or 2019 |
|  | MAJOR_COVERAGE_YN | Whether the patient is entitled to an increased reimbursement in YEAR X This is based on the social status of the patient (widow/widower, single parent, person with disabilities AND a low income ) and for all patients who are entitled to one of the following allowances: integration allowance for persons with disabilities, replacement income, allowance for help for the elderly, income guarantee for the elderly, subsistence minimum income, support from the Public Centre for Social Welfare. This can be used as proxy for socioeconomic vulnerability. |
|  | AGE10_CAT | Age of the patient: 40-49, 50-59, 60-69, 70-79, 80-89, 90-99, 100+ in YEAR X |
|  | DECEASED_YN | Whether patient died in YEAR X |
|  | SEX | SEX of the patient: male, female |
|  | COMMUNITY_CENTER_YN | Whether the patient is inscribed in a community center (which is a capitation system) in YEAR x |
|  | GP_COUNT | The number of GPs the patient consulted in YEAR X |
|  | GP_ORGANISATION_CAT | Whether the regular GP of the patient works as a solo-GP or in a group practice |
|  | CARE_REGION | Indicates to which care region patient’s residence belongs |
|  | POPDENSITY | Indicates the population density of patient’s residence. This information can be used to calculate the level of urbanization of the neighborhood |
|  | HOUSEHOLD_COMPOSITION | Indicates the composition of the patient's household |
|  | UNEMPLOYMENT_YN | Whether the patient had at least one day of unemployment in YEAR X |
|  | CHRONICAL_YN | Whether the patient is entitled to ‘statute of chronical illness’ |
|  | INVALIDITY_YN | Whether the patient had at least one day of invalidity for work in YEAR X |
|  | INCAPACITY_YN | Whether the patient had at least one day of primary incapacity for work in YEAR X |
|  | PROVINCE | In which province the patient lived in YEAR X: Antwerp, Limburg, East Flanders |
|  | STAT_SECTOR | To which statistical sector patient’s residence belongs (statistical sector is the basic territorial unit resulting from the subdivision of the territory of municipalities by STATBEL for the dissemination of its statistics at a finer level than the municipal level) |
|  | ONE_PARENT_FAM_YN | Whether the patient is a single parent |
|  | RESTHOME_YN | Whether the patient stayed 90 days or more in a nursing home in YEAR X |
|  | CARDIO_YN | Whether the patient is diagnosed with the pseudopathology* ‘cardiovascular disease’ |
|  | TROMBOSE_YN | Whether the patient is diagnosed with the pseudopathology* ‘thrombosis’ |
|  | HEART_DISEASE_YN | Whether the patient is diagnosed with the pseudopathology* ‘heart disease’ |
|  | COPD_3A01_YN | Whether the patient is diagnosed with the pseudopathology* ‘chronic obstructive pulmonary disease variant A’ |
|  | COPD_3B01_YN | Whether the patient is diagnosed with the pseudopathology* ‘chronic obstructive pulmonary disease variant B’ |
|  | ASTMA_04A01_YN | Whether the patient is diagnosed with the pseudopathology* ‘ASTHMA variant A’ |
|  | ASTMA_04B01_YN | Whether the patient is diagnosed with the pseudopathology* ‘ASTHMA variant B’ |
|  | MUCO_YN | Whether the patient is diagnosed with the pseudopathology ‘mucoviscidosis’ |
|  | CANCER_RT_YN | Whether the patient received reimbursement for radiotherapy in YEAR X |
|  | CANCER_MOC_YN | Whether the patient received reimbursement for a multidisciplinary oncology consultation in YEAR X |
|  | CANCER_CHEM_YN | Whether the patient received reimbursement for chemotherapy in YEAR X |
|  | DMT2_NO_INSUL_YN | Whether the patient is diagnosed with the pseudopathology* ‘Diabetes Mellitus without insulin’ |
|  | DMT2_INSUL_YN | Whether the patient is diagnosed with the pseudopathology* ‘Diabetes Mellitus with insulin’ |
|  | DMT2_CARDIO_YN | Whether the patient is diagnosed with the pseudopathology* ‘Diabetes Mellitus with cardiovascular disease(s)’ |
|  | ALZHEIMER_YN | Whether the patient is diagnosed with the pseudopathology* ‘Alzheimer's disease’ |
|  | HEMOPHILIA_YN | Whether the patient is diagnosed with the pseudopathology* ‘hemophilia’ |
|  | THYROID_GLAND_YN | Whether the patient is diagnosed with the pseudopathology* ‘thyronine disorder’ |
|  | KIDNEY_FAILURE_YN | Whether the patient is diagnosed with the pseudopathology* ‘renal failure’ |
|  | ORGAN_TRANSP_YN | Whether the patient is diagnosed with the pseudopathology* ‘organ transplantation’ |
|  | MS_YN | Whether the patient is diagnosed with the pseudopathology* ‘multiple sclerosis’ |
|  | HEPATITUS_YN | Whether the patient is diagnosed with the pseudopathology ‘chronic hepatitis B or C’ |
|  | HIV_YN | Whether the patient is diagnosed with the pseudopathology* ‘HIV’ |
|  | EPILEPSIE_NEURO_YN | Whether the patient is diagnosed with the pseudopathology* ‘epilepsy and neurotic pain’ |
|  | PARKINSON_YN | Whether the patient is diagnosed with the pseudopathology* ‘Parkinson's disease’ |
|  | PSYCHOSE_1401_YN | Whether the patient is 70 years old or older and diagnosed with the pseudopathology* ‘psychosis’ |
|  | PSYCHOSE_1301_YN | Whether the patient is younger than 70 years old and diagnosed with the pseudopathology* ‘psychosis’ |
|  | CROHN_YN | Whether the patient is diagnosed with the pseudopathology* ‘Crohn's disease, Ulcerosa Colitis, Psoriatric arthritis, or Rheumatoid arthritis’ |
|  | PANCREA_YN | Whether the patient is diagnosed with the pseudopathology* ‘exocrine pancreas disease’ |
|  | PSORIASIS_YN | Whether the patient is diagnosed with the pseudopathology* ‘psoriasis’ |
| TREATMENTS/CONULTS |  | Data delivered by the IMA healthcare dataset |
|  | TREATMENT_ID | Unique identifier of treatment or consult |
|  | PATIENT_ID | Unique identifier of the patient |
|  | NOMENCLATURE | Nomenclature number (for example: glucose measure, HbA1c measure, wound care, T2D educator consult, dietician consult, endocrinologist consult, ophthalmologist consult, selfcare material, etc.) |
|  | DATE | Date of the treatment or consult |
|  | PRACTITIONER_CAT | Identifies who provided the treatment or who was consulted by the patient |
|  | PRESCRIBER_CAT | Identifies who prescribed the treatment to the patient |
|  | INSTITUTION_CAT | Type of institution where the treatment consult took place: hospital, nursing home, psychiatric nursing homes, |
|  | COUNT | Identifies the number of treatments or consults |
|  | COSTCARE_ZIV | Cost of the treatment/consult: part that is covered by the health insurance (ZIV) |
|  | THIRD_PAYER_YN | Whether the treatment/consult is paid through the third payer system (when the patient only paid the co-payment) |
|  | COSTCARE_PERS | Cost of the treatment/consult: part that is paid by the patient (co-payment) |
|  | COSTCARE_SUPP | Cost of the treatment/consult: supplement |
|  | NIGHT_WEEKEND_YN | Indicates whether the treatment was provided/or the consult took place at night or during the weekend |
| PHARMACEUTICALS |  | Data delivered by the IMA Pharmanet dataset |
|  | PHARMA_ID | Unique identifier of medication |
|  | PATIENT_ID | Unique identifier of patient |
|  | PHARMA_PROD_CODE | Product code of medication |
|  | CKN_CODE | National code number (administrative number) |
|  | DATE_PRESCRIPTION | Date of the prescription of the pharmaceutical product |
|  | DATE_DELIVERY | Date of the delivery of the pharmaceutical product |
|  | ATC7 | The Anatomical Therapeutic Chemical Code of the pharmaceutical product (at level 7) |
|  | PHARMACIST_CAT | Who the medication provided to the patient |
|  | PRESCRIBER_CAT | Identifies who prescribed the medication to the patient |
|  | COSTMED_SUPP | Cost of the pharmaceutical product: supplement |
|  | COSTMED_PERS | Cost of the pharmaceutical product: part that is paid by the patient (co-payment) |
|  | COSTMED_ZIV | Cost of the pharmaceutical product: part that is covered by the health insurance (ZIV) |
| HOSPITALIZATION |  | Data delivered by the IMA about hospitalizations |
|  | HOSPITALIZATION_ID | Unique identifier of hospital stay |
|  | PATIENT_ID | Unique identifier of patient |
|  | HOSP_ADM | Identification of hospital admission |
|  | HOSP_TRANS | Identification of hospital transfer |
|  | STAY_CAT | Type of stay: hospital admission, one-day-admission, surgical one-day admission, admission to a psychiatric hospital, stay at a rehabilitation center |
|  | ADMISSION_DATE | Date of admission to the hospital |
|  | DICHARGE_DATE | Date of hospital discharge |
|  | DURATION | Length of the hospital stay in days |

**Pseudopathologies are algorithmically identified based on medication use of the patient*
