# Supplementary material for: Development and operationalization of a data framework to assess quality of integrated diabetes care in the fragmented data landscape of Belgium
Source: BMC Health Serv Res. 2022 Oct 18;22:1257. doi: 10.1186/s12913-022-08625-8 (PMC9578257; doi:10.1186/s12913-022-08625-8)
Supplement: Supplementary file 5 — Additional file 5. [file 12913_2022_8625_MOESM5_ESM.docx]

*Additional file 5. Figure: Multilevel design of the Integrated diabetes database*

**Patient level (individual level)** *(N = 7645)*

- Sociodemographics & pseudopaths
- Health care data (treatments/consults)
- Pharmaceutical data
- Clinical data (lab test results)

**GP-Practice level** *(N = 66)*

- Region: *Ghent, Antwerp, Kempen*
- Type of practice: *monodisciplinary & FFS, multidisciplinary & FFS, multidisciplinary & FFS*
- Organizational factors: *ACIC scores*
- Patient population characteristics: *volume, caseload, age and gender composition, …*
